# Ontogeny of the middle ear and auditory sensitivity in the Natterjack toad (*Epidalea calamita*)

**DOI:** 10.1242/jeb.244759

**Published:** 2022-11-11

**Authors:** Tanya Bojesen Lauridsen, Jakob Christensen-Dalsgaard

**Affiliations:** Department of Biology, University of Southern Denmark, Campusvej 55, DK-5230 Odense, Denmark

**Keywords:** Anuran, Development, Hearing, Middle ear

## Abstract

In most anuran amphibians, acoustic communication is of prime importance for mate localization and selection. The tympanic middle ear increases auditory sensitivity and directionality and is therefore expected to be favoured by natural selection. However, especially within the family of true toads (Bufonidae) there is a tendency for species to lose parts of the middle ear apparatus and consequently have a reduced sensitivity to high-frequency sounds (above 1 kHz). Part of the explanation for this may be that development of the middle ear is especially slow in bufonids, and thus the middle ear would be more likely to be lost or non-functional in paedomorphic species. However, a timeline of development of the middle ear has not been established previously. The goal of the present study was to investigate middle ear development in a toad species that has a well-known natural history and acoustic communication behaviour. We made a detailed study of anatomy and biophysics of the middle ear with measurements of auditory sensitivity across age in post-metamorphic natterjack toads (*Epidalea calamita*). The tadpoles and toadlets were raised in the laboratory, so their exact age was known, their auditory sensitivity was measured using auditory brainstem responses, and middle ear development and function were assessed by anatomical studies and laser vibrometry. We found that the developmental stage of the middle ear depends on the size of the toad rather than its age. The middle ear was functional at the earliest at a snout–vent length of 40 mm, which for these toads was around 500 days post-metamorphosis, close to the time of first reproduction. The functional, adult-like middle ear was shown to have 30 dB increased sensitivity to the dominant frequency of the mating call compared with sensitivities measured in newly metamorphosed individuals.

## Teaser

**Summary:** Natterjack toads show slow development of auditory sensitivity and maturation of the middle ear, which occur around 500 days post-metamorphosis; development seems to depend more on animal size than on age.

## INTRODUCTION

Acoustic communication is vital for mate recognition and localization in many species of animals, and most anuran amphibians call, often in choruses, to attract the attention of potential mates. Most anurans have inner ears with an auditory sensitivity peak at the frequency of their species-specific mating calls, essentially filtering out heterospecific calls of other frequencies and background noise (Wilczynski et al., 1984). In most species, call detection and localization are facilitated by the tympanic middle ear (Jaslow et al., 1988; Lombard and Straughan, 1974). The tympanic middle ear is often seen as a prerequisite for sensitive hearing of airborne sound, and has a similar configuration in non-mammalian tetrapods, caused by convergent evolution, as the middle ear developed independently in anurans, lizards, turtles and archosaurs (Bolt and Lombard, 1985; Clack, 1997).

However, especially within amphibians, the evolutionary history of the tympanic middle ear is complex. In the so-called ‘earless’ species (Jaslow et al., 1988; Pereyra et al., 2016; Womack et al., 2017) there are many instances of secondary loss of functionality of the middle ear (all species have functional inner ears, however). Loss of middle ear structures has occurred in many species within the family of true toads (Bufonidae) and based on the most recent phylogeny the last common ancestor of this group probably had a non-functional middle ear (Pereyra et al., 2016). Since then, middle ear structures have been regained in many bufonid branches, and even lost again in some species. This indicates that bufonids are prone to losing their middle ear structures through evolutionary processes (Pereyra et al., 2016). Because the structures of the middle ear system are the last to develop after metamorphosis (see below), changes in developmental rate (in the most extreme form, arrested development leading to paedomorphosis) has been suggested as a cause for the loss. Interestingly, larger cell size, caused by increased genome size, has been shown to decrease the developmental rate of nervous tissue, leading to secondary simplification. The effect has been shown in all amphibians, but is more prominent in salamanders and caecilians, because of their larger genomes (Roth and Walkowiak, 2015; Womack et al., 2019). Some bufonids have been shown to develop middle ear structures at an exceptionally slow rate. Hetherington (1987) showed that at the end of metamorphosis, only the stapes footplate was visible in *Bufo americanus*, and Womack et al. (2018) showed that the middle ear of *Rhinella horribilis* was still incomplete after 15 weeks of post-metamorphic development. Only one study has investigated developmental changes in ear morphology and the functional consequences for hearing sensitivity in bufonids (*Rhinella* sp. and *Rhaebo* sp.; Womack et al., 2016), showing a 14–27 dB increase in sensitivity from juveniles to adults that correlated with columellar ossification and thickening of the tympanic annulus.

### Structure and development of the anuran middle ear

**Fig. 1.**
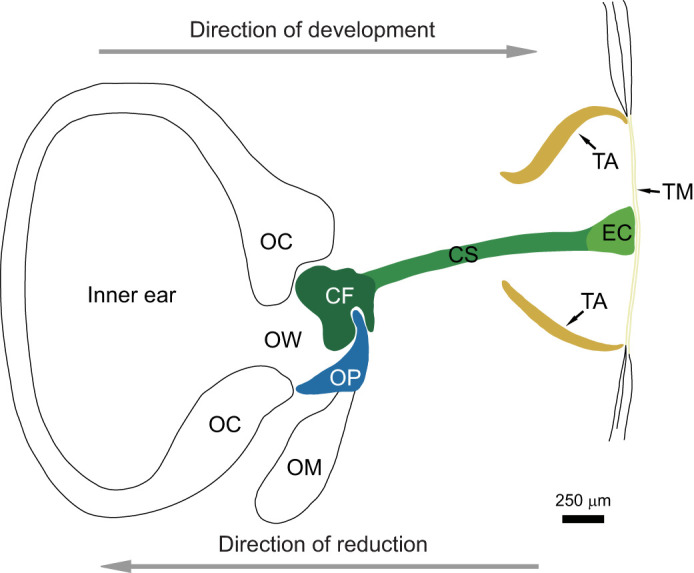
**Diagrammatic sketch of a fully functional tympanic middle ear at the final stage of metamorphosis in *Rana pipiens*.** During secondary reduction of middle ear structures, the most peripheral structures are lost before the more medial structures (arrow showing direction of reduction). CF, columellar footplate; CS, columellar shaft; EC, extra-columella; FO, fenestra ovalis, oval window; OC, otic capsule; OM, opercularis muscle; OP, operculum; OW, oval window; TA, tympanic annulus; TM, tympanic membrane. The operculum is formed first (blue); the columella is then formed in three parts: columellar footplate (first), then columellar shaft and extra-columella (last) (dark green to light green). The tympanic annulus (TA) is formed before the tympanic membrane (TM). Modified from Hetherington (1987).

Fully functional tympanic middle ears in terrestrial anurans (Fig. 1) consist of the tympanic membrane, supported around the edge by the cartilaginous tympanic annulus, and the columella, which distally connects to the tympanic membrane through the extra-columella, and proximally connects to the otic capsule through the columellar footplate, which takes up the anterior half of the oval window. The development of the tympanic system [studied in detail in *Rana temporaria* (Villy, 1890; Vorobyeva and Smirnov, 1987) and in the aquatic frog *Xenopus laevis* (Bever et al., 2003; Vorobyeva and Smirnov, 1987)] starts from the oval window and extends in a proximal to distal direction. A mesenchymal condensation, which becomes the columellar footplate, forms at the anterior edge, after the operculum has formed at the posterior edge. As the footplate starts to chondrify, the shaft of the columella begins to form. In most species, the extra-columella then starts forming from a separate centre of chondrification underneath the skin, distal from the columellar shaft (Hetherington, 1988), but in some species (e.g. *Alytes obstetricans* and *Bufo regularis*; Banbury and Maglia, 2006; Sedra and Michael, 1959) the extra-columella is formed as a further extension of the columellar shaft. At the end of development of the tympanic middle ear, the shaft has ossified, while the footplate and extra-columella usually remain cartilaginous. Concurrent with the formation of the columella, the middle ear cavity and tympanic annulus also develops. The last event in anuran middle ear morphogenesis is the formation of the tympanic membrane through several histological changes to the integument suspended in the tympanic annulus (Hetherington, 1988; Smirnov, 1991). Some species have fully developed middle ear structures at the end of metamorphosis or shortly thereafter (Shofner and Feng, 1981), while other species have delayed development of these structures (Hetherington, 1987; Womack et al., 2018) and some remain partially or completely earless (Hetherington and Lindquist, 1999). The reduction of the middle ear apparatus follows the reverse order of development (Hetherington, 1987; Sedra and Michael, 1959; Vorobyeva and Smirnov, 1987) (see arrows in Fig. 1). This means that the tympanic membrane is not present if the columella and tympanic annulus are absent, but these last two structures can be present without the tympanic membrane (Pereyra et al., 2016; Womack et al., 2016).

The amphibian ear contains a unique movable element, the operculum (OP in Fig. 1), which is connected to the suprascapula by the opercularis muscle. The system may function in sensing seismic vibrations (Hetherington, 1985; Kingsbury and Reed, 1909), possibly also in extra-tympanic hearing (Hetherington, 1985; Lombard and Straughan, 1974), and it has been suggested to protect the inner ear from high-amplitude movements during breathing or vocalization (Mason and Narins, 2002). The operculum starts to form when the forelimbs start to develop (Gosner stage 27; Hetherington, 1987; Sedra and Michael, 1959), and when the forelimbs emerge (Gosner stage 40) the opercularis system is fully developed. The timing of the development of the opercularis system shows very little variation between anuran species, contrary to the development of the tympanic middle ear, which has been shown to vary greatly across the entire order of anuran amphibians (Hetherington, 1987; Jaslow et al., 1988; Pereyra et al., 2016).

### Natural history of the natterjack toad

The natterjack toad, *Epidalea calamita* (Laurenti 1768), has a tympanic middle ear, and is grouped with other ‘eared’ bufonid species in the most recent phylogeny (Pereyra et al., 2016). Their nightly choruses, which are of extremely high amplitude (loudest European anuran), can be heard from up to 2 km away on clear nights (Sinsch, 1997), and may have evolved as a consequence of this species' preference for breeding in ephemeral ponds (Beebee, 1979). The life history of this species is well described in many aspects (Beebee, 1979; Sinsch, 1998), including the onset of their breeding behaviour. Leskovar et al. (2006) reported that first-time breeders in Germany and Spain are usually 3 years old (age determined by skeletochronology; Smirina, 1994), but that some early breeders reproduce after their second hibernation (11.4% of males, 8.1% of females, minimum snout-to-vent length, SVL, of 55 mm). Denton and Beebee (1993) reported that toads in British and continental European populations that had surpassed the apparent minimum size threshold of 43 mm SVL often postponed their first reproduction for a year. From this, they concluded that age was the defining factor of when sexual maturity was attained.

The present study was undertaken to compare development of the morphology of the middle ear in this species [investigated by microcomputed tomography (µCT) scans and differential staining for bone and cartilage], with development of functionality, assessed as auditory sensitivity measured by auditory brainstem responses, and biophysical properties of the middle ear, measured by laser Doppler vibrometry. Our hypothesis was that the delayed development of the middle ear structures is reflected in reduced sensitivity to sound.

## MATERIALS AND METHODS

### Animal care

We used 45 natterjack toads (*Epidalea calamita*) collected as eggs or tadpoles, and hatched in 2014 (18 individuals), 2016 (13 individuals) and 2017 (10 individuals). Four adult toads were caught in the wild (2018) and released after auditory brainstem response and vibrometry experiments. The age (in days post-metamorphosis, dpm) and size (SVL in mm) were recorded for each animal. Groups were created to have at least three animals of the same age to ensure the basis for statistical analysis, with more groups in the younger generations as we expected changes to be more rapid early after metamorphosis. Size groups were created *post hoc* when we saw that this factor could also be a predictor for the maturation of the middle ear. Data from wild-caught animals were used as a reference for a fully functional middle ear, but as their exact age was unknown, they were excluded from all statistical models. For further details on each individual, see Table S1.

The animals were kept under laboratory conditions in paludariums on a 13 h:11 h light:dark cycle at 22°C and with a room humidity of 55%. They were fed size-appropriate food items such as springtails, fruit flies and mealworms. To imitate natural conditions, the toads were induced to hibernate during the winter months. They were kept in boxes filled with a mixture of sand, topsoil and leaves, and they were left outside for approximately 1 week to experience the natural diurnal temperature fluctuations. The boxes were moved to a fridge and kept at 5°C until spring, at which point they were moved back outside for a week to ‘wake up’ the toads before they were returned to their laboratory paludariums. To identify the individuals between experiments, some were kept in isolation and some were recognized by their individual belly patterns. The experiments were approved by the Danish National Animal Experimentation Board (Dyreforsøgstilsynet). The toads are protected by Danish law, and we had permission from the Danish Nature protection agency (Skov- og Naturstyrelsen) to collect eggs, tadpoles and adult toads.

The animals were subjected to procedures in the following order: (1) auditory brainstem response to sound (ABR), (2) auditory brainstem response using vibration as stimulation (vABR), (3) laser Doppler vibrometry to measure the sound-induced vibration of the jaw, the eardrum (eardrum region), the centre of the skin overlying the lung and the hindleg, (4) euthanasia, (5) µCT scanning, and (6) differential staining for bone and cartilage (see Table S1 for details).

### Anaesthesia

Prior to experiments, the animals were anaesthetized by brief immersion in a 0.2% tricaine methane sulfonate (MS-222, Sigma-Aldrich) solution buffered to neutral pH with sodium bicarbonate. During immersion, the animals were under constant observation and when there was no reflex response to toe pinching, the toads were lightly rinsed in running tap water, before being placed in the experimental setup. To counter bias in sensitivity thresholds due to fluctuations in anaesthetic levels, we randomized the sequence of tested frequencies. We also monitored the anaesthetic level by intermittently running a click stimulation series. For more in-depth discussion, see Lauridsen et al. (2021).

### ABR and vABR

The experimental setup used for these experiments was identical to that described in Lauridsen et al. (2021). However, the electrodes used differed between larger and smaller individuals, as follows. In bigger animals, three electrodes (disposable subdermal needle electrodes, 27 gauge, 12 mm, Rochester Electro-Medical Inc., Lutz, FL, USA) were placed subcutaneously, (1) dorsal to the left ear (inverting), (2) dorsal to the brainstem (non-inverting), and (3) in the distal front leg (ground reference). For smaller animals, custom electrodes were made from silver wires (silver wire, 76.2 μm bare diameter, 139.7 μm Teflon-coated diameter; A-M Systems Inc., Carlsborg, WA, USA), as per Goutte et al. (2017). These electrodes were placed similarly, (1) subcutaneously dorsal to the left ear (inverting), by making a small hole with a needle and then inserting the electrode, (2) in the distal nostril (non-inverting), and (3) in the cloaca (ground reference). The electrodes were connected to the system as described in Lauridsen et al. (2021).

#### Sound stimulation

The animals were stimulated with 25 ms tone bursts, shaped by a cos^2^ envelope, averaged over 2000 samples at frequencies between 200 and 5000 Hz (200, 400, 600, 800, 1000, 1300, 1600, 1900, 2300, 2700, 3100, 3500, 4000, 4500 and 5000 Hz) at intensities from 25 to 110 dB re. 20 µPa, in increments of 5 dB.

#### Vibration stimulation

The loudspeaker was replaced by a vibration exciter (Brüel & Kjær Type 4809, Nærum, Denmark). The experimental animal was placed on a platform on the vibration exciter in two positions: (1) dorso-ventral stimulation – animal sitting in a natural upright position, by positioning the front legs beneath the chest; and (2) lateral stimulation – animal lying on its side with the electrode-connected ear directly on the vibration table, held in place using Play-Doh. These two positions were chosen to illustrate naturally occurring movements: (1) dorso-ventral movement of the toad from ground vibration, i.e. footsteps of an approaching researcher, and (2) lateral vibration, i.e. the animal being ‘pushed’ and ‘pulled’ by sound waves hitting the animal from the side, which would occur when one amphibian is calling towards another individual. The setup was calibrated using an Accelerometer Type 4381 (calibrated by a Calibration Exciter type 4294), and a Nexus Conditioning Amplifier Type 2692-A-0S4 (all three pieces of equipment from Brüel & Kjær, Holte, Denmark). Vibration stimulus duration (25 ms) and frequencies (200–5000 Hz) were equal to those of the sound experiment and intensities ranged from 50 to 150 dB re. 10 µm s^−2^ tested in 5 dB increments.

### Laser Doppler vibrometry

The anaesthetized toad(-let) (for details, see Table S1) was placed on a platform, in an upright, natural position, at the centre of an anechoic room. The sound-induced vibration of four areas of interest – (1) the jaw right beneath the eye, (2) eardrum (eardrum region), (3) the skin overlying the centre of the lung and (4) the hindleg – was measured to investigate whether the entire animal was being moved by the soundwaves or if only parts of the animal were responsive to sound. Twelve speakers (JBL IG, Northridge, CA, USA) were placed in a circle (diameter 2 m) with 30 deg intervals. Two microphones were placed close to the animal: one (½ inch G.R.A.S. microphone, Brüel & Kjær) just above the head, used for calibration, and a probe microphone (type 4182, Brüel & Kjær), recording the sound close to the area of interest (jaw, eardrum, lung and leg). The probe microphone was mounted on a micro-manipulator fixed to a stand on the ground for easy re-positioning between tests. The laser was placed perpendicular to the direction the toad was facing, pointing at the left lateral side, and it was fixed to a manipulator mounted on a heavy tripod, to reduce vibrations from the floor. To increase laser reflections, reflective microbeads (glass beads, diameter: 50 µm, alumina-silicate microspheres, Omya AG, Oftringen, Switzerland) were placed on the area of interest. The animal was stimulated with 175 ms frequency sweeps (0–8 kHz) at selectable levels up to 84 dB SPL re. 20 µPa. The sweeps were generated using custom-written software and Tucker-Davis system 2 hardware (Tucker-Davis Technologies, TDT, Alachua, FL, USA). The signals were deconvoluted by dividing the spectrum of the sweep with the transfer function (measured during calibration) of the individual speakers, before sending them to the loudspeakers. Sweeps were emitted from each loudspeaker separately (10 averages), using a customized switching device. The sound was recorded (22 kHz sample rate, 8192 samples) using the probe while simultaneously measuring the vibrations using an OFV-5000 laser Doppler vibrometer with an OFV-505 sensor (Polytec, Waldbronn, Germany). The analog laser signal was digitized using an A–D converter (AD2, TDT). During trials, the laser signal was continuously manually monitored using a dual trace oscilloscope (15 MHz, Leader LBO-514A) and a vibrometer controller (Polytec OFV-5000). Stimulation and recording were controlled by custom-written software (*DragonQuest* by J.C.-D.). Because of availability of facilities, only selected individuals were tested using this method of measurement.

### Euthanasia

The animals were euthanized by overdosing in MS-222 immersion (15–30 min depending on size) and were then preserved in 70% ethanol.

### µCT scanning

µCT scans of the otic region of some of the toads (for details, see Table S1) were obtained using a vivaCT40 scanner (Scanco Medical, Brüttisellen, Switzerland). The applied electrical potential across the X-ray tube was 70 kV with a current of 0.114 mA and with an isotropic voxel size of 10.5–19 µm based on the size of the specimen (and hence the field of view of the scanner). Each voxel was measured once with an exposure time of 300 ms. 3D reconstructions were obtained using the scanner’s custom software. Because of availability of facilities, only selected individuals were tested using this method of measurement.

### Differential staining for bone and cartilage

Some of the specimens (for details, see Table S1) were differentially stained for bone (Alizarin Red S) and cartilage (Alcian Blue) using a protocol adapted by Nicholas Ditzel (Molecular Endocrinology Department, SDU) from Inouye's (1976) original protocol.

### Recording of natterjack call

A calling male natterjack toad was recorded in Tarup-Davinde, Funen, Denmark, in May 2017 using an Olympus LS10 digital recorder (sample rate 44 kHz). The recorder was calibrated using a ½ inch microphone (Brüel & Kjær). The recorded call can be seen in Fig. S2.

### Data analysis

#### Grouping of animals

To test the effect of age and size (SVL) on the functionality (hearing threshold) of the middle ear, the animals were assigned to two different groups according to these two parameters (Table S1).

#### ABR

The sensitivity thresholds for audiograms were determined by visual inspection of response curves cross-referenced with objectively determined thresholds, where peaks with a signal to noise ratio above 0.1 were accepted as a response (Lauridsen et al., 2021).

#### Laser Doppler vibrometry

We calculated transfer functions from the laser vibrometry experiments by dividing the vibration spectra (measured by the laser) by the sound spectra recorded by the probe microphone.

#### Effect of age and size on sensitivity thresholds

As age and size are probably correlated, we wanted to investigate the effects of each to understand the correlation between age and/or size and hearing threshold at 1600 Hz. We explored these effects using a model selection framework in which we tested four linear regression models: two univariate models (threshold predicted by age and size, respectively), one bivariate model (threshold predicted by age with the additional effect of size, and vice versa) and one interaction model (does the effect of age depend on the effect of size, and vice versa?).

All data analysis was carried out using R 3.6.2 and MATLAB R2019b. For the model selection approach, Akaike's information criterion (AIC) was used. It was calculated using the AIC function in R (packages: dplyr, ggplot2), which uses the formula AIC=2*K*−2log*L*, where *K* is the number of independent variables and *L* is the log-likelihood estimate (the likelihood that the model could have produced the observed *y*-values). Surface plots were created using SigmaPlot 12.5. µCT scan illustrations were made using the custom software of the scanner; outlines were added in Inkscape. The middle ear illustration in Fig. 1 was created using Inkscape, modified from Hetherington (1987).

## RESULTS

**Fig. 2.**
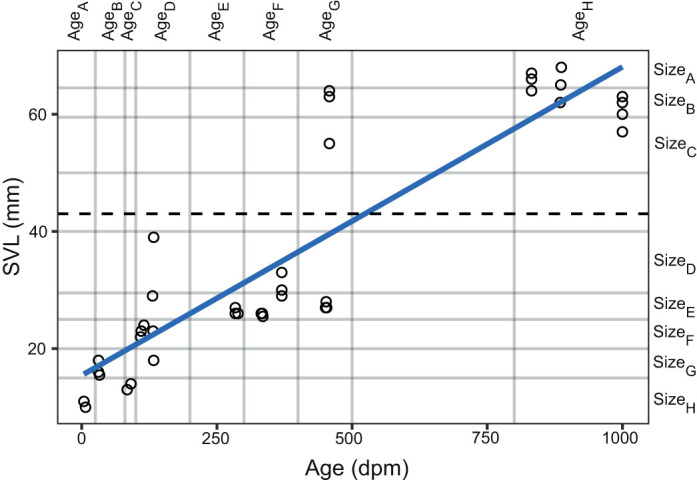
**Size of the toads as a function of their age.** Horizontal grey lines indicate size groups (snout-to-vent length, SVL); vertical grey lines indicate age groups. The horizontal dashed line is the minimum size threshold (43 mm SVL) for breeding reported by Denton and Beebee (1993). The blue line is the fitted linear regression (*R*^2^=0.8068). dpm, days post-metamorphosis.

In total, 45 animals were used in this study over the course of 4 years. One had gonads full of eggs when it was euthanized, all the wild-caught animals were chorusing males, and the rest were not sexed. The age of the wild-caught animals is not known, but they must have reached the reproductive stage, as they were engaging in reproductive behaviours when caught. The youngest animal tested was 4 dpm (11 mm SVL), the oldest (with recorded age) was 887 dpm (68 mm SVL). The smallest animal measured 10 mm SVL (7 dpm), and the largest was 68 mm (887 dpm; Fig. 2). There was a gap of no animals from approximately 500 to 800 dpm, and 40–50 mm SVL. There was great variability of the eardrums (or eardrum region) upon inspection by the naked eye. In the small animals, they were un-developed and hence could not be seen. Some mature adults had a relatively clear outline of the eardrums, but in others it was difficult to discern the border around the edge. The eardrums of all individuals seemed undifferentiated from the surrounding skin.

Not unexpectedly there was a significant (*R*^2^=0.8068, *P*<0.01) correlation between age and size (Fig. 2). However, we did also see that some individuals grew more rapidly than their peers, essentially creating age groups, where some individuals were twice as large (18–39 mm SVL and 27–63 mm SVL, respectively) as other individuals of the same age and from the same egg clutch (groups age_D_ and age_G_, respectively).

### ABR and vABR

To establish whether the best predictor for thresholds is age or size, we took a model selection approach. The model selection indicated highest support for a univariate model using size as a predictor for threshold (Table 1), with a significant effect (β=−0.38, *P*<0.01). Age (β=−0.02, *P*<0.01) was also significantly correlated with the thresholds, but the magnitude of the effect was much lower than that of size. Adding age to the model (bivariate model) did not improve the predictive performance, and we conclude that the similar AICs for models A, C and D are largely a result of the effect of size. Based on this, we used size as a predictor for thresholds from this point and on, but comment on both when appropriate.

**Table 1.**
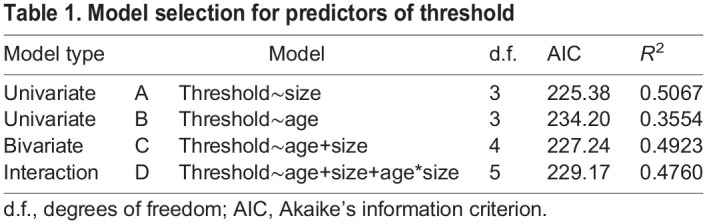
Model selection for predictors of threshold

**Fig. 3.**
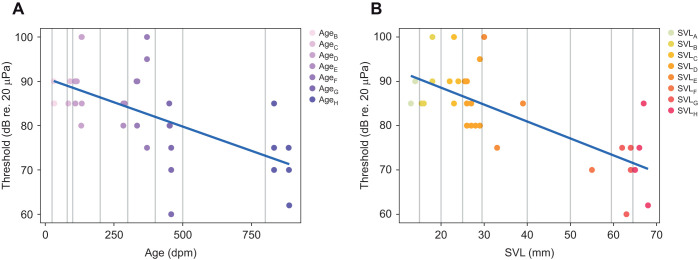
**Auditory thresholds at the mating call frequency (1600 Hz) as a function of age and size.** Blue lines indicate the fitted linear regression: (A) age, *R*^2^=0.3554; and (B) SVL, *R*^2^=0.5067. Grey vertical lines indicate the groups. Group age_A_ is not included in A, as this group had no responses to this frequency.

**Fig. 4.**
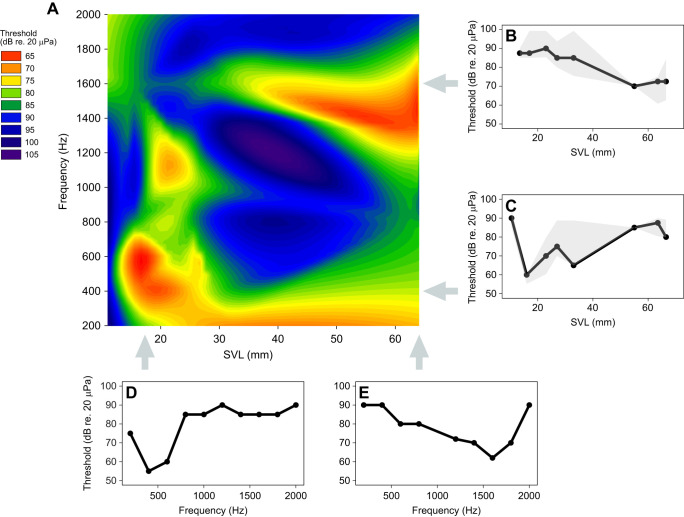
**Changes in hearing threshold as a function of size and age.** (A) Hearing threshold (dB re. 20 µPa, colour scale) as a function of size (SVL) and frequency. Data were smoothed using linear interpolation and boxcar averaging. (B) Median sound threshold (with 95% confidence intervals, CI) at 1600 Hz for the SVL groups. (C) Median sound threshold (with 95% CI) at 400 Hz for the SVL groups. (D) Audiogram of a small (15 mm SVL) and young (33 dpm) individual. (E) Audiogram of a large (68 mm SVL) and old (887 dpm) individual.

Fig. 3 shows the increase of sensitivity to 1600 Hz as a function of age (A, *R*^2^=0.3554, *P*<0.01) and size (B, *R^2^=*0.5067, *P*<0.001), and their fitted regression lines. There were no responses to sounds above 3100 Hz, and only a few animals responded to frequencies between 2000 and 3100 Hz. Both the youngest (age_A_) and the smallest (SVL_A_) group only responded to low frequencies (200, 400 and 600 Hz), and the thresholds of these responses were relatively high (85–95 dB re. 20 µPa). The most sensitive individual (15.5 mm SVL, 33 dpm, age_B_ and SVL_B_) detected the 400 Hz stimulus down to 55 dB SPL. As the animals grew older and bigger, they started responding to higher frequencies, including the frequency of their mating call, 1600 Hz (Fig. 4). The individual (63 mm SVL, 458 dpm, age_G_ and SVL_G_) that was most sensitive to the call frequency detected it at 60 dB SPL, and in general sensitivity to this frequency was seen in toads larger than 40 mm SVL.

Auditory brainstem responses to dorso-ventral vibration stimulation (vABR) showed a sensitivity threshold down to 65 dB re. 10 µm s^−2^ to low-frequency stimuli (Fig. S1) of 200–600 Hz for all age and size classes. There were no responses to stimuli above 2700 Hz. Sensitivity to higher frequencies increased as the animals got older and bigger, but the thresholds at these frequencies were never as low as for the low frequencies. There was a sensitivity peak at 2000 Hz in the oldest/biggest animals (Fig. S1). The animals seemed to be less sensitive to lateral vibration stimulation, but the recordings were inconclusive and were hence excluded.

### Laser Doppler vibrometry

**Fig. 5.**
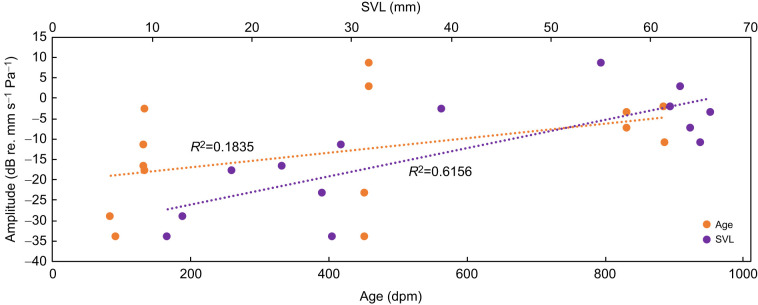
**Acoustic responsiveness to 1600 Hz as a function of age and size.** Dotted lines indicated the fitted linear regression for age (orange, *R*^2^=0.2197) and size (purple, *R*^2^=0.6291).

The relationship of eardrum vibration as a function of age and size is shown in Fig. 5. When we used age as a predictor, the increase in the responsiveness of the eardrum to 1600 Hz was less pronounced than when using size as the predictor. We measured vibrations of the jaw and hindleg to assess the sensitivity to tympanic versus extratympanic sound reception. The induced vibrations of the jaw were similar to, but of smaller amplitude than, the response of the eardrum. The results from the hindleg were similar to the control measurements of the platform on which the toad was placed.

**Fig. 6.**
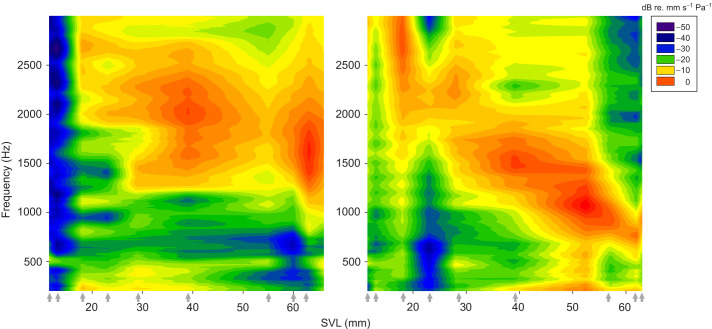
**Vibration velocity surface plots of eardrum (eardrum region) (left) and lung (right) as a function of size and frequency.** Grey arrows indicate the size of the sampled animals. Eardrum vibration amplitude is shown by the colour scale.

The acoustic responsiveness of the eardrum changed dramatically with size (Fig. 6, left). The eardrum (eardrum region) of the smallest toads did not vibrate when stimulated with sound. This changed as the toads grew bigger and a sensitivity peak emerged around 2000 Hz, and in the largest individuals the peak had moved to around 1600 Hz, corresponding with the peak frequency of the mating call. The response of the lung also changed as the toads grew bigger (Fig. 6, right). In the small animals, the lung responded with high-frequency vibrations up to 3000 Hz. During development, when the lung volume increases, the peak frequency decreases and in the largest animals the lung resonates at around 1000 Hz.

**Fig. 7.**
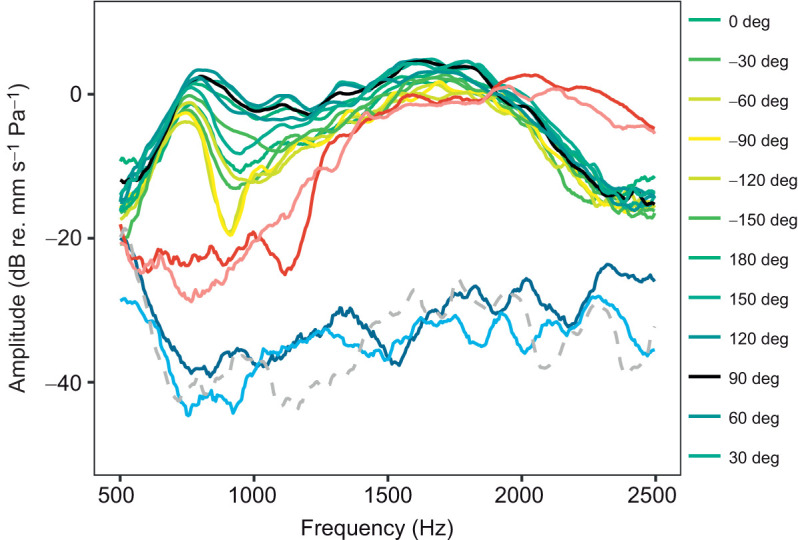
**Vibration velocity spectra (transfer functions) of the eardrum in three differently sized toads.** The response of the eardrum to sounds from 12 directions is shown for an adult, wild-caught individual, of size 62 mm SVL. Black represents the ipsilateral sound direction (90 deg), yellow represents the contralateral sound direction (−90 deg) and green represents intermediate directions, as shown in the key (darker shading: ipsilateral, lighter shading: contralateral). The lung resonance frequency coincides with the peak below 1000 Hz. The largest amplitude is seen at 1500 Hz. The differences in amplitude between the 12 speakers around 1000 Hz are caused by the directionality of the tympanic middle ear system. The response is also shown for a 39 mm SVL individual, where red represents the ipsilateral sound direction (90 deg) and pink represents the contralateral sound direction (−90 deg), and for a 28 mm SVL individual, where dark blue represents the ipsilateral sound direction (90 deg) and light blue represents the contralateral sound direction (−90 deg). The grey dashed curve shows the sound-induced vibration of the platform.

The bigger animals also exhibited directionality to sound (Fig. 7). At the mating call frequency (1600 Hz), the difference in amplitude between the ipsilateral and contralateral stimulation was approximately 6 dB. Just below 1000 Hz, the difference between ipsilateral and contralateral stimulation was around 20 dB.

### µCT scans

**Fig. 8.**
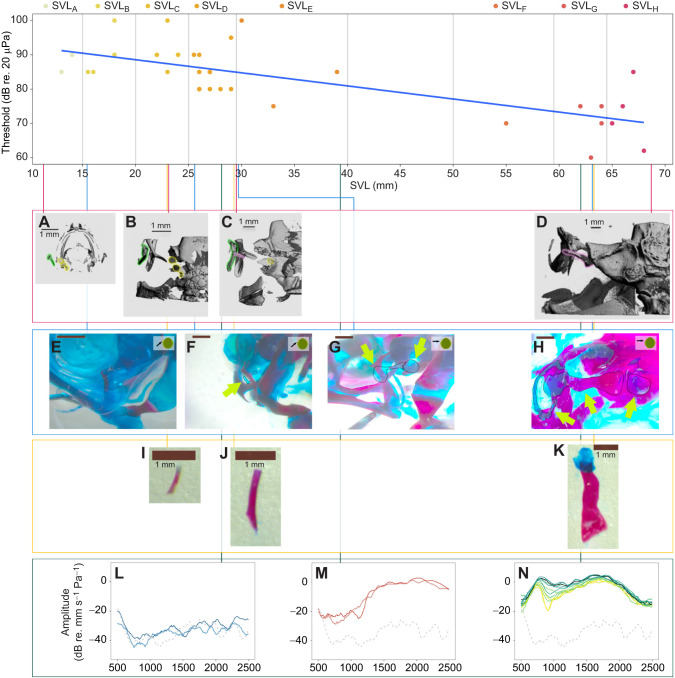
**Summary of key findings.** Top: increase of hearing sensitivity to the dominant frequency of the mating call (1600 Hz). Red box: dorsal view µCT scans of the left side otic region of four individuals: (A) 11 mm SVL/4 dpm, (B) 23 mm SVL/110 dpm, (C) 29 mm SVL/131 dpm, (D) 68 mm SVL/887 dpm. Otoliths are outlined in yellow, the squamosal is outlined in green and the columella is outlined in pink. Blue box: differential staining of cranial regions of four individuals: (E) 15.5 mm SVL/33 dpm, no trace of any middle ear structures; (F) 25.5 mm SVL/335 dpm, a small aggregation of blue cells, will probably form the extra-columella, and a tiny columella shaft; the operculum (not outlined) is visible to the right of the outlined columella; (G) 29 mm SVL/370 dpm, thin extra-columella, connected to the shaft and the footplate; the operculum is also outlined; (H) 63 mm SVL/458 dpm, fully developed middle ear, from left: tympanic annulus, extra-columella, columella shaft, columella footplate and operculum. Yellow box: differentially stained columellae of three individuals: (I) 23 mm SVL/132 dpm, (J) 29 mm SVL/131 dpm and (K) 63 mm SVL/458 dpm. Bone is red, cartilage is blue. Green arrows point to middle ear structures. Green box: vibration velocity spectra (transfer functions) of the eardrum in three toads: (L) 28 mm SVL/453 dpm, dark blue is the ipsilateral sound direction (90 deg), light blue is the contralateral sound direction (−90 deg); (M) 39 mm SVL/133 dpm, red is the ipsilateral sound direction (90 deg), pink is the contralateral sound direction (−90 deg); (N) the response of the eardrum to sounds from 12 directions in an adult, wild-caught individual (SVL 62 mm); black curve is the ipsilateral sound direction (90 deg), yellow is the contralateral sound direction (−90 deg). The peak below 1000 Hz is caused by resonance in the lung; the peak above 1500 Hz is caused by vibration of the eardrums. The differences in amplitude between the 12 speakers around 1000 Hz are caused by the directionality of the tympanic middle ear system. The grey dashed curve is the sound-induced vibration of the platform.

The µCT scans (Fig. 8A–D; Fig. S3) show the stage of ossification of the middle ear and other cranial structures. In the smallest toad (11 mm; Fig. 8A), the only sign of an ear is the three otoliths in each of the inner ears. The otic capsule is beginning to show in the second toad (23 mm; Fig. 8B), but the otic capsule is still not fully closed, and the three otoliths are still visible. Part of the columella can be seen in the third toad (29 mm; Fig. 8C), and it looks more or less like a small tube. The ossification of the columella starts at the distal part and proceeds medially towards the oval window. The otoliths are still visible inside the inner ear from a lateral view, but there is a clear difference in ossification of the otic capsule between Fig. 8B and C, where the otic capsule is partially covering the otoliths. Scans of an individual with 39 mm SVL were similar to those from the individual with 29 mm SVL (Fig. 8C). In the largest specimen (68 mm; Fig. 8D), the otic capsule has ossified completely, and the columella spans the middle ear cavity. The distal part is elongated, and the medial part widens out. There was no sign of an ossified tympanic annulus at any stage.

### Differential staining for bone and cartilage

The smallest individuals showed no staining for either bone or cartilage within the otic region (Fig. 8E). In an individual with a SVL of 23 mm (Fig. 8I), a small tube-like columella can be seen at the most distal part of the otic region. It is nestled within the squamosal (Fig. S4). Fig. 8I,J,K shows three columellae from toads of different sizes. All three are ossified, but the two small columellae have cartilaginous ends. The cartilaginous (blue) part of the large columella is the extra-columella, which was attached to the tympanic membrane. Another cartilaginous element, the operculum, is visible at least from 300 dpm (Fig. 8F).

Results of µCT scans and differential stains are summarized in Table S2, and pictured in Fig. 8A–D; Figs S3 and S4.

## DISCUSSION

The development of high-frequency sensitivity and the directionality of the middle ear apparatus is significantly correlated with the size of the individual and the corresponding developmental stage. We show that the natterjack toads up to a size of approximately 40 mm are insensitive to the frequencies of their own mating call. This corresponds well with the minimal size threshold (43 mm SVL) for breeding reported by Denton and Beebee (1993). Toads of this size are at least 500 dpm, in individuals with rapid growth (Fig. 2). This time span for development of a functional middle ear is considerably longer than that reported for other bufonid species. The middle ear of the common African toad [*Sclerophrys* (*Bufo*) *regularis*] is probably functional 1 year post-metamorphosis, suggested by the adult-like morphology of the middle ear at this stage (Sedra and Michael, 1959). However, these anatomical studies are not yet supported by functional investigations. A timeline for ear development has not been established prior to this study, but the ‘threshold size’ of 40 mm is comparable to the distinction between juvenile and adult size bufonids (comparable in size to natterjack toads) studied by Womack et al. (2016). Growth rates are probably slower in the natterjack toads, which live in temperate zones, compared with the tropical species studied by Womack et al. (2016) and Womack et al. (2018), which will delay the development of the middle ear.

### Hearing sensitivity and its consequences for mating call detection

As acoustic communication in the natterjack toad is well described, it is possible to assess the consequences for mating call detection. The laser vibrometry measurements show that the mature tympanic ear increases the sensitivity in the frequency band of the call by more than 30 dB (Fig. 7) and also generates directionality in this frequency region. According to the inverse square law (6 dB/distance doubling), a 30 dB increase in sensitivity corresponds to a 2^5^=32 times [30 dB divided by 6 (dB/distance doubling)=5], increase in detection distance under optimal conditions in open habitats. The loudest natterjack call published, recorded by Arak (1988), was reported to be 108 dB SPL at 0.5 m distance. The lowest ABR threshold we measured was 60 dB SPL at the mating call frequency, and as ABR thresholds are usually approximately 20 dB higher than the lowest single-unit neural or behavioural thresholds (Lauridsen et al., 2021), we estimate that the toads would respond to around 40 dB SPL at the mating call frequencies. A 40 dB SPL threshold in the adult toads would result in a detection distance of more than 1 km, a distance where Arak (1988) also observed females to respond phonotactically to the calls of males.

The earliest breeders in *E. calamita* are 2 years old (Leskovar et al., 2006), so sexual maturity may be coincident with the maturation of the middle ear, at least in individuals with rapid growth. Factors that influence growth are crowding of tadpoles and the availability of nutrition in the breeding pools. This in turn has an effect on the toadlets (Denton and Beebee, 1993), and as size significantly influences the timing of development of auditory sensitivity, it is possible that maturation of the middle ear of the smaller individuals can be delayed by as much as a year. With this result, we challenge the conclusion of Denton and Beebee (1993) that age is the dominant parameter of when the toads can engage in breeding behaviour.

### Earlessness

The prolonged development before reaching maturity in this ‘eared’ toad species (the natterjack toad) also suggests why earless bufonid species are common: if the delayed development is a general trait in bufonids, they are already earless for a large part of their life in nature, and a slight shift in the timing of development, for example by lowered ambient temperature, could potentially lead to complete earlessness. Many other factors than delayed development could lead to earlessness (reviewed by Pereyra et al., 2016). For example, miniaturization (many earless species are small) would make the eardrum less sensitive, if its size decreases allometrically. Sensitivity measured as maximal vibration amplitude increases with the square of the radius – so allometric miniaturization would naturally lead to a less-sensitive, stiffer eardrum, and for very small species, the benefits of having a tympanic ear in terms of sensitivity might be negligible. Low-frequency sensitivity does not change during the different developmental stages, so this sensitivity is probably caused by extra-tympanic sound reception. The simplest mechanism is that the toad is vibrated by the sound wave, i.e. pushed and pulled by the incident sound pressure, probably stimulating the sensory cells by inner-ear fluid inertia (Capshaw et al., 2022; Christensen-Dalsgaard et al., 2022 preprint). Also, the operculum develops earlier than the tympanic middle ear (Fig. 8G), providing a possible extratympanic input to the inner ear (Capshaw et al., 2022).

We compared the auditory brainstem thresholds of the natterjack toads with the sensitivity of the eared (*Rhinella tacana*, *R. alata*, *R. leptoscelis*, *R. spinulosa*, *R. horribilis* and *Rhaebo haematiticus*) and earless (*R. arborescandens*, *R. festae*, *R. yunga* and *Osornophryne guacamayo*) toad species studied by Womack et al. (2017), using the same programme and a similar setup. At low frequencies (300–400 Hz), the natterjacks of our study were less sensitive (lowest sensitivity 55 dB SPL) than the South American species (lowest sensitivity for both eared and earless, 45 dB SPL). At the high-frequency sensitivity peak, our adult natterjacks (60 dB SPL at 1600 Hz) fell in between the eared (45 dB SPL at 1500 Hz) and the earless (64 dB SPL at 1500 Hz) species’ lowest thresholds. Womack et al. (2017) also showed that size (ranging from approximately 20 to 110 mm SVL in this study) did not influence sensitivity thresholds.

### Morphological changes

The µCT scans and differential stains show that the ossification of the columella starts between SVL sizes of 23 and 29 mm. At 29 mm, the distal part is visible on the µCT scans, showing that the columella ossifies in the reverse direction to the direction of initial development (Fig. 1). The middle ear of the differentially stained individual (SVL 23 mm; Fig. 8I) is more similar to the µCT scan in Fig. 8C (SVL 29 mm), and a small, ossified part of the columella can be observed at the distal portion of the middle ear. These differences are probably caused by differences between individuals. In the µCT scan of the individual that has reached adult size (SVL 68 mm; Fig. 8D), the columellar shaft is complete and the otic capsule is closed and fully ossified. The formation of the tympanic middle ear is one of the last events in the morphogenesis of an anuran amphibian, because it is linked to changes in the mechanism of the jaw from the larval state to adult function, a change that takes place at the end of metamorphosis. The drift of the palato-quadrate enables the tympanic annulus to grow from this cartilage (Gaupp, 1893) and if the process is delayed, the time frame in which the overlying skin (that in functional middle ears becomes the tympanic membrane) is physiologically active may be passed, and this may cause the skin spanning the tympanic annulus to remain thick (Smirnov, 1991). This seems to be the case for the natterjack toads, as a cartilaginous tympanic annulus is present in the older and larger individuals, but the skin overlying the tympanic membrane is not differentiated. The same is seen in *Bufo bufo*, where the tympanic membrane remains covered in thick skin and the tympanic annulus is only fully chondrified when the toad reaches sexual maturity (Smirnov, 1991).

It would have been interesting to follow the development of sound sensitivity in the same animals, but for obvious reasons there was a conflict between wanting to track the neurophysiological and biophysical changes in the same individuals and the desire to investigate the morphological conditions (differential staining) of the different age and size groups. Also, our toads were reared under laboratory conditions with fixed temperature and an abundance of food. These conditions may have favoured faster growth than natural conditions, which could lead to the experimental animals maturing faster than their wild counterparts. The data gaps (age: 500–800 dpm and size: 40–50 mm SVL) could be filled by collecting toads of appropriate sizes in the field, testing their hearing sensitivity in the laboratory, and releasing them back to the wild. By sampling a toe from each individual before release, their age could be determined via skeletochronology (Smirina, 1994), a method that has proven reliable for natterjack populations with seasonal changes (Tejedo et al., 1997). It would probably be helpful to investigate the molecular mechanism behind the developmental process, by either *in situ* hybridization or immunohistochemistry. The sections from these experiments could also be used to investigate the morphology and development of the inner ear.

## Supplementary Material

10.1242/jexbio.244759_sup1 Supplementary information
