# Supplementary material for: Ontogeny of the middle ear and auditory sensitivity in the Natterjack toad (Epidalea calamita)
Source: J Exp Biol. 2022 Nov 11;225(21):jeb244759. doi: 10.1242/jeb.244759 (PMC9687611; doi:10.1242/jeb.244759)
Supplement: Supplementary information [file jexbio-225-244759-s1.pdf]

Table S1. Overview of experimental animals. Each group contains animals that were subject to experiments at the same age. Metamorph.: end of metamorphosis. d.p.m.: days post metamorphosis. Head: width of the head measured at the eardrums. Vib. d/v: vibration stimulation in a dorsal/ventral direction (test animal in a natural upright position). Vib. L.: vibration stimulation in a lateral direction (test animal placed on the side). X's indicate that the animal in that specific row had been subject to the method indicated by the column. Empty rows indicate NAs. Orange rows contains animals from 2014, blue rows animals from 2016, green rows animals from 2017 and grey rows wild-caught animals. "X" indicates that the animal has been subject to the experimental method. "V" (µCT) indicates that the animal was scanned, but that the scan was incomplete, and re-scans were not possible, because the animal had been stained or dissected in the meantime. Individuals EC17d and EC17e were removed from the data-analysis as they did not wake up from anaesthesia.

ID	age group	SVL group	Metamorph. d.m.y	Mass [g]	Head [mm]	Age [d.p.m.]	SVL [mm]	Auditory brainstem response: Sound	Vib. d/v	Vib. L	Laser	µCT	Dissection	Stain
EC14a	B	B	25.05.14		6.0	31	18	X				X	X	
EC14b	B	B	25.05.14		5.5	31	16	X				X		X
EC14c	B	B	25.05.14		5.5	33	15.5	X				X		X
EC14d	D	C	25.05.14		9.0	109	22	X	X					
EC14e	D	C	25.05.14		9.0	110	23	X	X			X	X	
EC14f	D	C	25.05.14		9.0	115	24	X	X			X		X
EC14g	E	D	25.05.14		8.5	284	26	X	X			X		
EC14h	E	D	25.05.14		9.5	284	27	X	X			X		X
EC14i	E	D	25.05.14		10.0	289	26	X	X			X		X
EC14j	F	D	25.05.14		9.0	332	26	X	X			V		X
EC14k	F	D	25.05.14		9.5	334	26	X	X					
EC14l	F	D	25.05.14		9.5	335	25.5	X	X			X		X
EC14m	F	E	25.05.14		13.0	370	33	X	X			V	X	
EC14n	F	E	25.05.14		10.5	370	30	X	X			V	X	
EC14o	F	D	25.05.14		11.0	370	29	X	X					X
EC14p	H	G	25.05.14	43.91	24.0	885	62	X	X		X	X		X
EC14q	H	H	25.05.14	33.27	22.0	886	65	X	X		X			
EC14r	H	H	25.05.14	36.58	22.0	887	68	X	X		X	X		X
EC16a	D	E	25.06.16	6.94	14.0	133	39	X	X	X	X	X		X
EC16b	D	D	25.06.16	6.47	14.0	131	29	X	X	X	X	X		
EC16c	D	C	25.06.16	1.08	8.0	132	23	X	X	X	X	X		X
EC16d	D	B	25.06.16	0.49	7.0	133	18	X	X	X	X			X
EC16e	G	D	25.06.16	2.01	10.0	451	27	X	X	X	X			
EC16f	G	D	25.06.16	2.22	10.5	452	28	X	X	X	X	X		X
EC16g	G	D	25.06.16	1.77	10.0	453	27	X	X	X				
EC16h	G	F	25.06.16	19.91	17.0	458	55	X	X	X	X	X		
EC16i	G	G	25.06.16	28.20	22.0	458	63	X	X	X	X	X		X
EC16j	G	G	25.06.16		22.0	458	64	X	X	X	X			
EC16k	H	H	25.06.16		24.0	832	66	X	X		X			
EC16l	H	H	25.06.16		24.0	832	67	X	X		X	X		
EC16m	H	G	25.06.16		23.0	832	64	X	X		X	V		X
EC17a	A	A	20.06.17	0.14	4.0	4	11	X	X	X		X		X
EC17b	A	A	20.06.17	0.22	4.5	7	13	X	X	X		X		X
EC17c	A	A	20.06.17	0.12	4.0	7	10	X	X	X				
~~EC17d~~			~~20.06.17~~	~~0.06~~	~~3.5~~	~~14~~	~~8~~	~~X~~	~~X~~	~~X~~	-	~~V~~	-	-
~~EC17e~~			~~20.06.17~~	~~0.10~~	~~3.5~~	~~14~~	~~10~~	~~X~~	~~X~~	~~X~~	-	-	-	~~X~~
EC17f	C	A	20.06.17	0.23	4.5	84	13	X	X	X	X			
EC17g	C	A	20.06.17	0.24	5.0	84	13	X	X	X		X		X
EC17h	C	A	20.06.17	0.25	4.5	91	11.5	X	X	X	X	X		
EC17i	C	A	20.06.17	0.33	5.0	91	14	X	X	X				
EC17j	C	A	20.06.17	0.35	5.0	91	14	X	X	X				X
ECWa	H	F	wild-caught	18.5	21.0	1000	57	X	X					
ECWb	H	G	wild-caught	30.9	22.0	1000	62	X	X					
ECWc	H	G	wild-caught	23.1	19.0	1000	60	X	X					
ECWd	H	G	wild-caught	20.0	19.0	1000	63	X	X					

Table S2. Morphological results of differential stains for bone (red) and cartilage (blue) and µCT scans (showing ossified structures).

Animal	SVL (mm)	Age (d.p.m.)	Differential stain	µCT
EC14c	15.5	33	No structures	No structures
EC14e	23	110	-	No structures
EC14f	24	115	-	No structures
EC14g	26	284	-	Small distal shaft
EC14i	26	289	Aggregation of blue cells at the site of the tympanic membrane/extra-columella	Small distal shaft
EC14L	25.5	335	Aggregation of blue cells at the site of the tympanic membrane/extra-columella, very thin ossification of columella shaft.	Small distal shaft
EC14 o	29	370	Small ossified columella shaft	Small coluemlla
EC14p	62	885	-	Fully ossified shaft and footplate
EC14r	68	887	-	Fully ossified shaft and footplate
EC16a	39	133	-	Small distal shaft
EC16b	29	131	-	Small distal shaft
EC16c	23	132	Small ossified columella shaft	Ossified shaft
EC16d	18	133	Small part in the middle of the columella has ossified, blue aggregation at the eardrum	No structures
EC16f	28	452	Blue eardrum/annulus<br>Blue extra-columella connecting shaft (ossified) and eardrum<br>Visible operculum	Ossified shaft
EC16h	55	458		Fully ossified shaft
EC16i	63	458	Fully ossified footplate and shaft, chondrified extra-columella and tympanic annulus.	Full shaft and footplate
EC16L	67	832	-	Full shaft and footplate
EC17a	11	4	-	No structures
EC17e	10	14	No structures	No structures
EC17g	13	84	Aggregation of blue cells at the site of the tympanic membrane/extra-columella	No structures
EC17h	11.5	91	-	No structures
EC17j	14	91	Aggregation of blue cells at the site of the tympanic membrane/extra-columella	No structures

Journal of Experimental Biology • Supplementary information

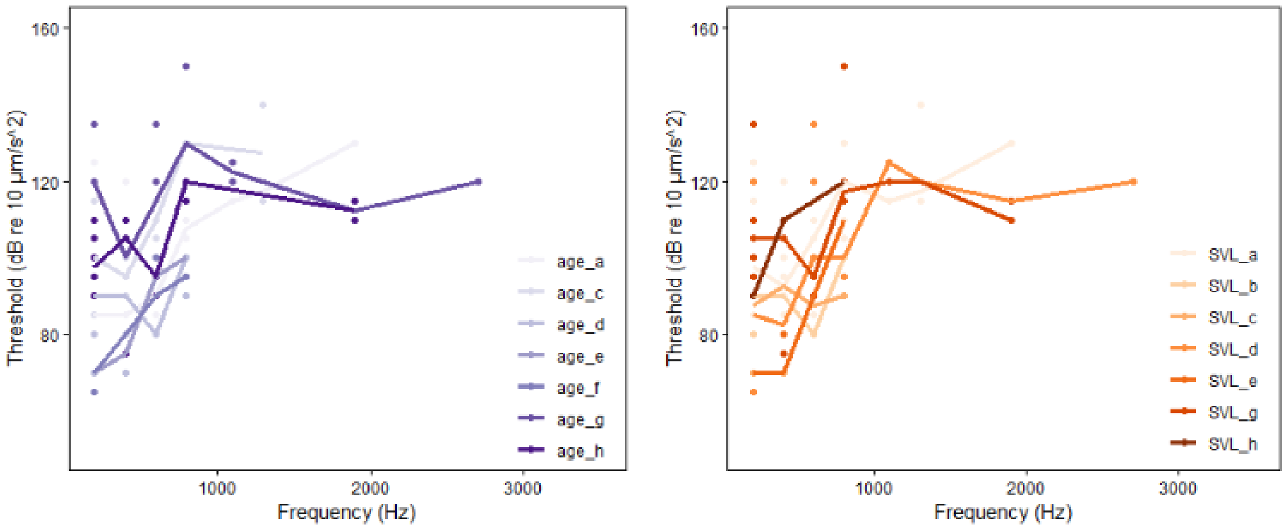

Fig. S1. Auditory brainstem responses to dorso/ventral vibration stimulation for each age (left) and size (right) group. Lines are medians of each group respectively. Lighter colours indicate younger and smaller animals, darker colours indicate older and bigger animals.

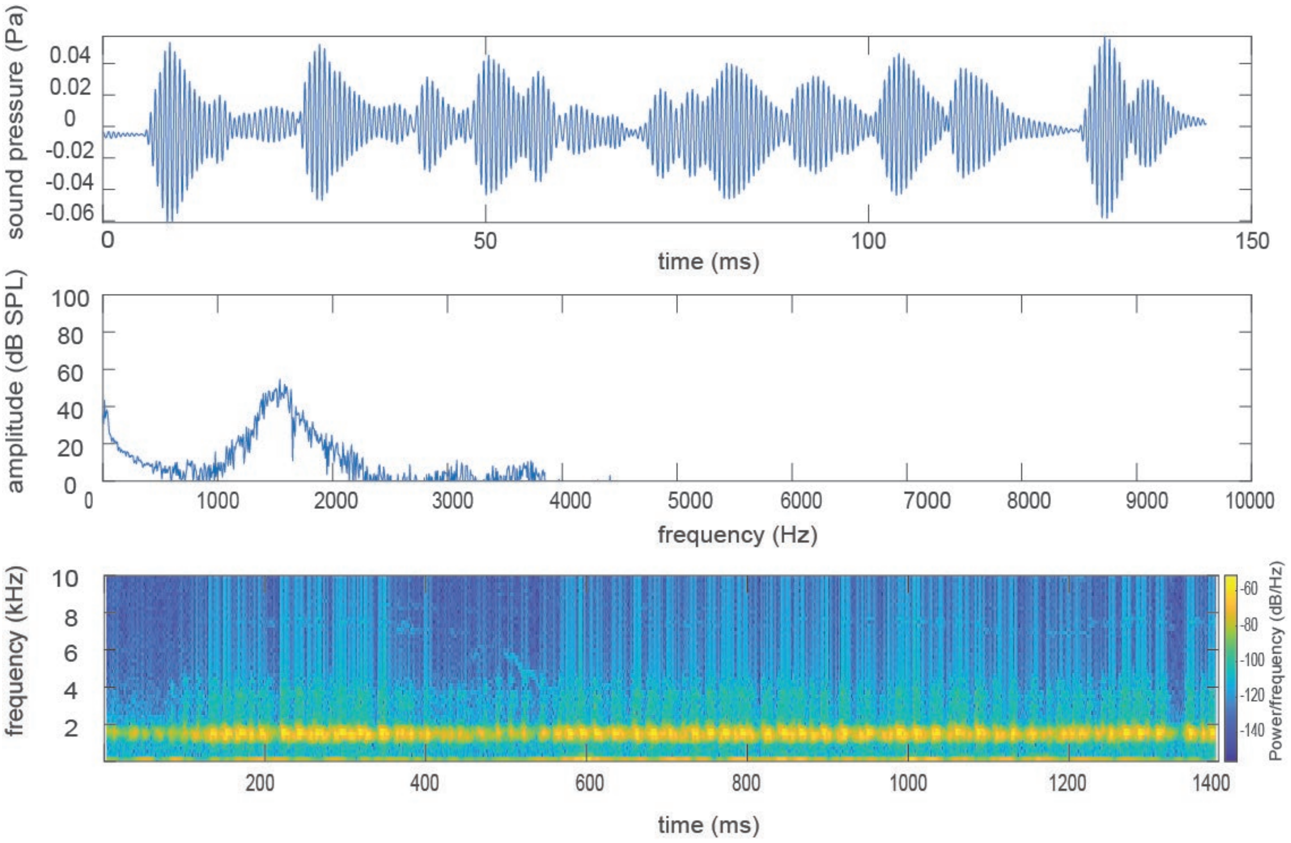

Fig. S2. Oscillogram, frequency spectrum and spectrogram of a field recording (JCD) of a calling natterjack toad. Spectrogram settings: FFT size: 512, overlap 500/512 98%, 512 points Hamming window. From this recording the source level of a single male calling was calculated to be 82 dB re. 20 µPa at a distance of 1 m.

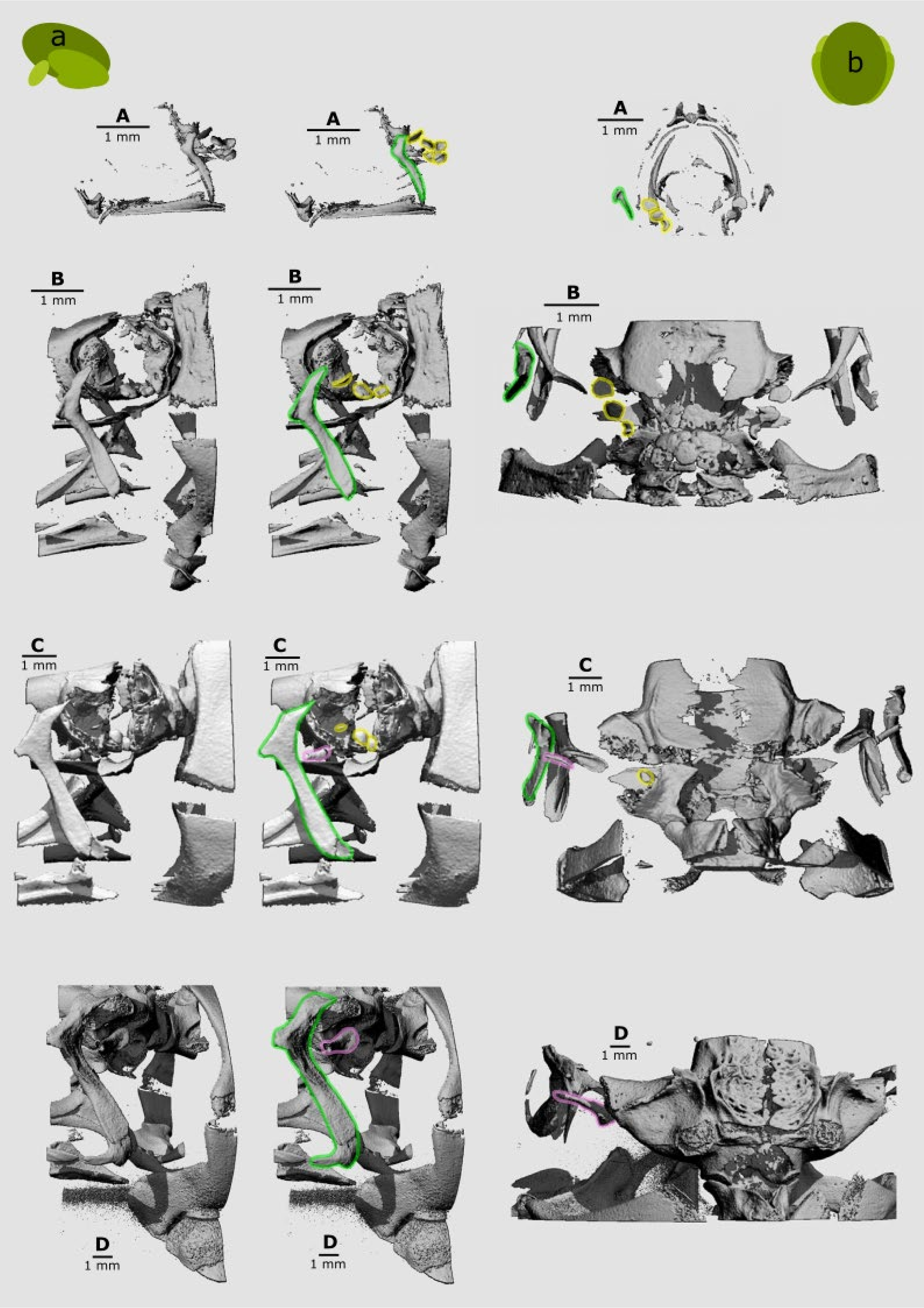

Journal of Experimental Biology • Supplementary information

Fig. S3. μCT scans of natterjack toad cranial regions at four developmental stages. A: 11 mm, B: 23 mm, C: 29 mm, D: 68 mm. Otoliths are outlined in yellow, the squamosal is green, and the columella is pink. Note the differences in the ossification of the otic capsule. The scans on the left and in the middle are lateral view of the same left ear region (oriented as the diagrammatic toad a in the top left corner), without and with outlines respectively. Scans on the right are a dorsal view of the ear region (oriented as the diagrammatic toad b in the top right corner), comparisons with and without outlines can be made between the left and the righthand side of the scan.

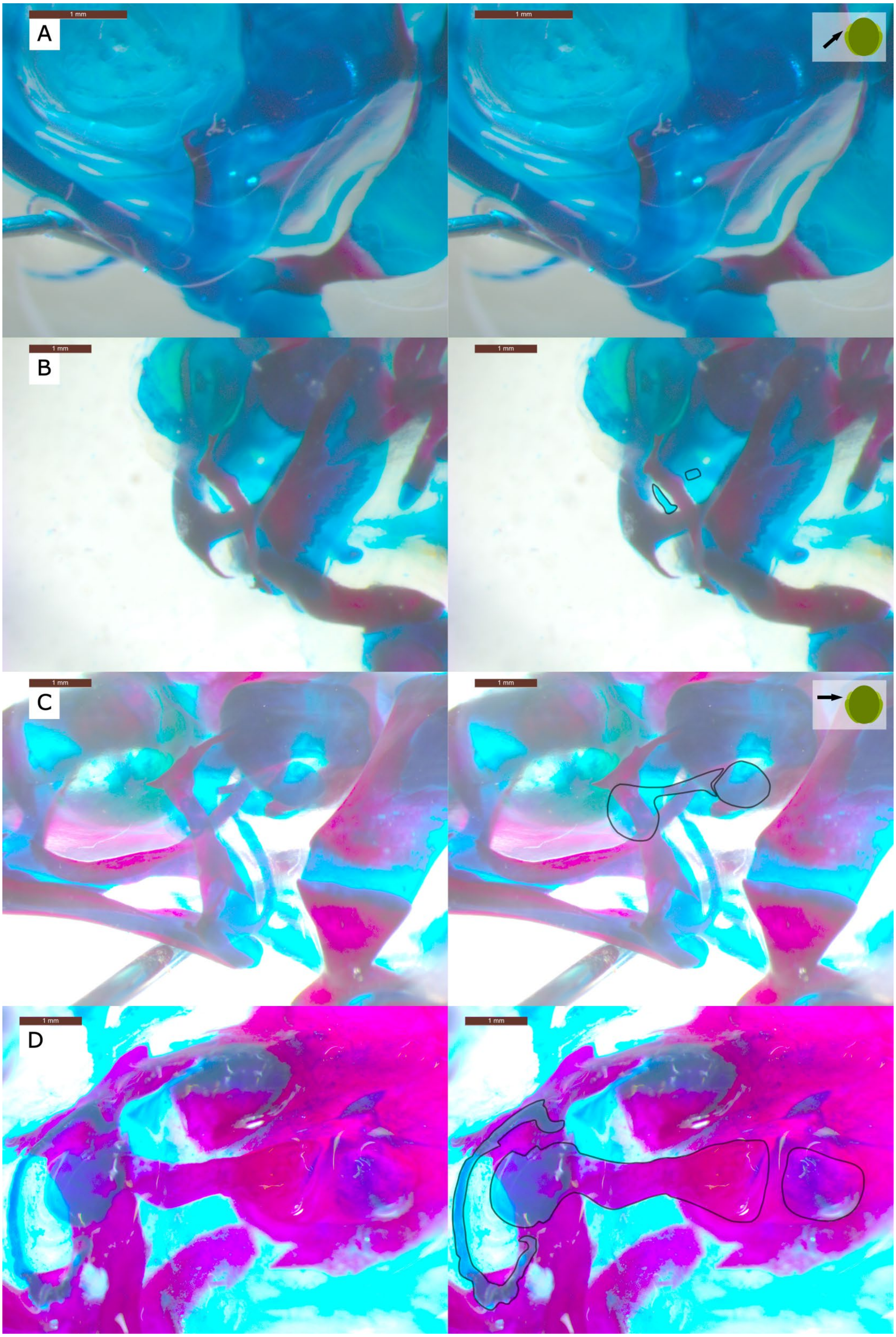

Fig. S4. Differential stained cranial regions of natterjack toads at four developmental stages: A: 15.5 mm, no trace of any middle ear structures. B: 25.5 mm, a small aggregation of blue cells (will probably form the extra-columella) and a tiny columella shaft. C: 29 mm, thin extra-columella, connected to the shaft and the footplate. Operculum is also outlined. D: 63 mm, fully developed middle ear, from left: tympanic annulus, extra-columella, columella shaft and columella footplate. Operculum is also outlined. Each picture is displayed twice, on the right outlines are added around the middle ear structures. Pictures were taken from a lateral view, in A and B with an angle pointing forward (see small diagram top right), in C and D perpendicular to the head (see small diagram), to enable best lighting conditions to capture the structures.

Journal of Experimental Biology • Supplementary information
